# Supplementary figures and images for: Genomic Variation in Korean japonica Rice Varieties
Source: Genes (Basel). 2021 Oct 30;12(11):1749. doi: 10.3390/genes12111749 (PMC8623644; doi:10.3390/genes12111749)

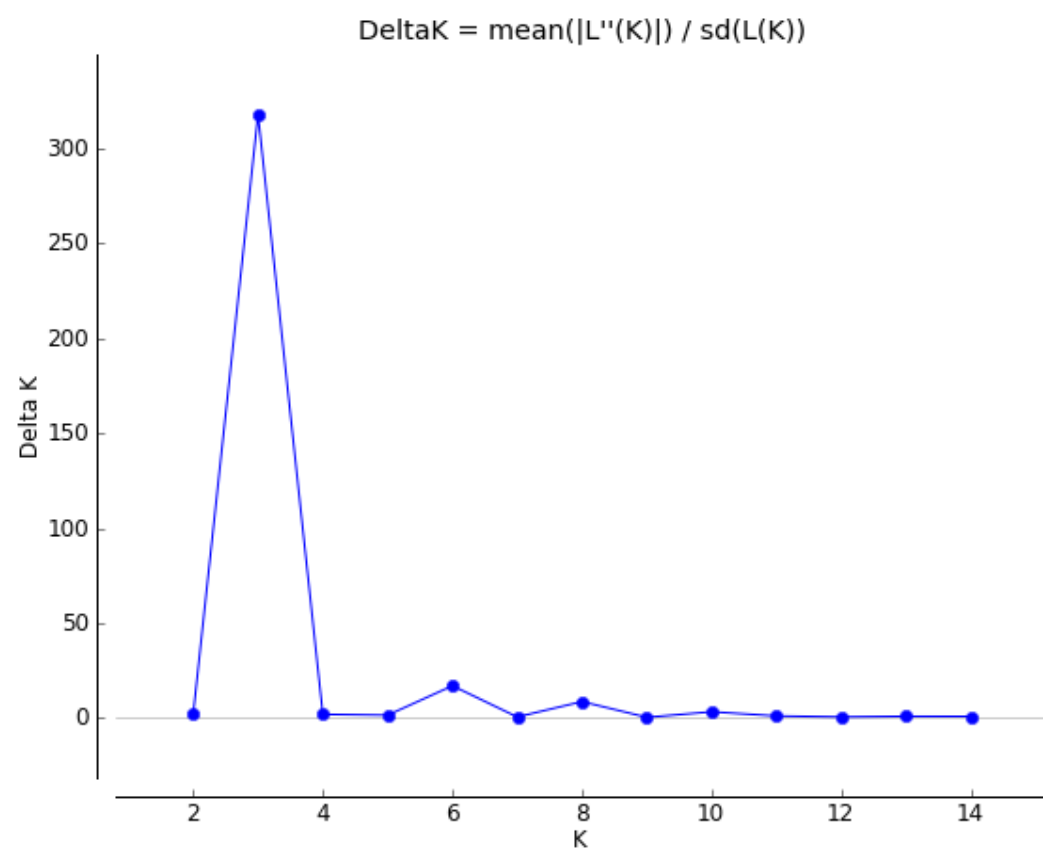

Figure S1. Estimation of Population Using LnP(D) Derived  $\Delta K$  for  $K$  from 1 to 15.

Supplement: Supplementary file 1 [file genes-12-01749-s001.zip › Figure_S1.pdf]
